# Phenolic Compounds and Triterpenes in Different Olive Tissues and Olive Oil By-Products, and Cytotoxicity on Human Colorectal Cancer Cells: The Case of Frantoio, Moraiolo and Leccino Cultivars (*Olea europaea* L.)

**DOI:** 10.3390/foods10112823

**Published:** 2021-11-16

**Authors:** Pujun Xie, Lorenzo Cecchi, Maria Bellumori, Diletta Balli, Lisa Giovannelli, Lixin Huang, Nadia Mulinacci

**Affiliations:** 1 Institute of Chemical Industry of Forest Products, CAF, Nanjing 210042, China; pujunxie@caf.ac.cn (P.X.); l_x_huang@163.com (L.H.); 2 National Engineering Laboratory for Biomass Chemical Utilization, Nanjing 210042, China; 3 Key and Open Laboratory on Forest Chemical Engineering, SFA, Nanjing 210042, China; 4 Key Laboratory of Biomass Energy and Material, Nanjing 210042, China; 5 Co-Innovation Center of Efficient Processing and Utilization of Forest Resources, Nanjing Forestry University, Nanjing 210037, China; 6 Department of NEUROFARBA, Nutraceutical and Pharmaceutical Section, University of Florence, Via Ugo Schiff 6, Sesto Fiorentino, 50019 Florence, Italy; lo.cecchi@unifi.it (L.C.); maria.bellumori@unifi.it (M.B.); diletta.balli@unifi.it (D.B.); 7 Department of NEUROFARBA, Pharmacology and Toxicology Section, University of Florence, Viale Pieraccini 6, 50139 Florence, Italy; lisa.giovannelli@unifi.it

**Keywords:** olive seeds and shells, olive leaves and branches, olive pomace, secoiridoids, pentacyclic triterpenoids, phytocomplex, colon cytotoxicity

## Abstract

Phenolic and triterpenoid compounds of the olive tree are recognized as having a key role in health promotion, thanks to their multiple protective actions in humans. To expand the source of these bioactive compounds, the phenolic and triterpenoid profiles of leaf, branch, destoned fruit, destoned pomace, shell, seed, and extra virgin olive oil from the *Frantoio*, *Leccino*, and *Moraiolo* olive cultivars were simultaneously characterized by HPLC-DAD-MS. Overall, 43 molecules were quantitated and expressed on the obtained dry extracts. Oleuropein was mainly concentrated in branches (82.72 g/kg), fruits (55.79 g/kg), leaves (36.71 g/kg), and shells (1.26 g/kg), verbascoside (4.88 g/kg) in pomace, and nüzhenide 11-methyl oleoside (90.91 g/kg) in seeds. Among triterpenoids, which were absent in shells, the highest amount of oleanolic acid was found in olive leaves (11.88 g/kg). HCT-116 colorectal cells were chosen to assess the cytotoxicity of the dry extract, using the phytocomplex from Frantoio, which was the richest in phenols and triterpenoids. The IC_50_ was also determined for 13 pure molecules (phenols and terpenoids) detected in the extracts. The greatest inhibition on the cell’s proliferation was induced by the branch dry extract (IC_50_ 88.25 μg/mL) and by ursolic acid (IC_50_ 24 μM). A dose-dependent relationship was observed for the tested extracts.

## 1. Introduction

Olive tree (*Olea europaea* L.) is predominantly distributed in Spain, Italy, and Greece, but is spreading throughout the world, including in China [1]. Olive oil sector takes the lead in the global market-share of high-quality oil, particularly the extra virgin olive oil (EVOO), thanks to its great health-promoting and sensory attributes catering to the healthy diet for modern consumers. Olive oil is a milestone of the Mediterranean diet; its production achieved annually 2.86 million tons in the period between 2005/06 and 2017/18, accounting for approximately 2.0% of all edible oils [2]. Italy owns most of the olive cultivars present worldwide (approximately 600 cultivars), representing 25% of the current olive germplasm [3], and is reckoned as a golden district for olive oil production, yielding high-quality virgin olive oils owing to suitable Mediterranean climate conditions and fine management in the mill. Among the numerous cultivars from Italy, Frantoio, Leccino and Moraiolo were regarded as high-quality cultivars with suitable oil yield and successfully transplanted to emerging countries (i.e., China, India, South Africa, and Australia).

In the wake of pruning practices, harvesting and olive milling processing, an annual world production of several millions of tons of olive fruit, olive cake, olive pomace, olive branches, olive leaves and olive stone (shell and seed) has been estimated [4]. Sometimes, several olive by-products are used for animal feeding (e.g., olive cake, olive pomace, olive leaves), or as fuel (e.g., olive branch, olive stone), but often they are simply directly thrown away, burned or grinded and scattered on the field, potentially causing environmental damages. Overall, the management of these by-products represents an increasing cost for producers, which are responsible for their management and elimination. At the same time, these olive by-products are rich in high-added value molecules such as phenolic compounds belonging to secoiridoids, lignans, flavonoids, phenolic acids and simple phenolic alcohols, and triterpenoids as maslinic, oleanolic and ursolic acids and erythrodiol, thus constituting a vast resource of bioactive molecules [5,6,7,8]. Since the demand for safe and healthy natural molecules has markedly increased along with the consumers’ preference for natural additives in food and cosmetics [9], various efforts have been devoted to valorizing these resources [10]. Certainly, it is vital to clearly grasp the composition of the raw materials to define suitable processes to recover high-value substances, which can constitute ingredients for the formulation of food supplements, cosmetics, and pharmaceuticals. Indeed, most of these molecules exhibited bio-pharmacological properties as antioxidant, antimicrobial, anticarcinogenic, anti-diabetes, prevention of cardiovascular disease, radioprotective and anti-inflammatory [11,12]. Nowadays, olive leaf extracts are widely accepted for global consumers in the nutraceutical market because of some modern health-promoting claims and traditional treatment of other ailments [13]. The crude olive pomace, constituted by olive pulp and olive stone, can be used to recover the so-called crude olive pomace oil by mean of solvent extraction. However, more and more often, olive by-products have been evaluated for identifying new potential sources of bioactive phytochemicals, as phenolic compounds, and triterpenoids [14].

The phenolic compounds profile consistently varies in the different olive tree tissues (e.g., leaves, twigs, or small branches) and olive oil production by-products, mainly depending on the presence of specific secoiridoids and glycosylated molecules. Leaves encompass a high content of phenolic compounds, such as oleuropein and verbascoside, along with several flavonoids as luteolin and apigenin. The olive pomace has been found to be rich in hydroxytyrosol and verbascoside [15]. Among the *Olea europaea* L. tissues, the seeds and branches are those less studied, with salidroside and nüzhenide only isolated in olive seeds [15]. As for the terpenoids, olive leaves and pomace contain high levels of maslinic and oleanolic acids. For instance, the amounts of oleanolic acid ranged from 3.0 to 3.5% in the leaf, followed by major levels of maslinic acid and minor levels of ursolic acid, erythrodiol and uvaol [7].

Colorectal cancer (CRC), one of the most life-threatening epithelial malignancies, is epidemically the third widespread around the world with a high fatality rate [16]. The Mediterranean diet has been recognized as protective against CRC because it is rich in health promoting molecules, including those derived by virgin olive oil. Some bioactive ingredients from olive tissue and olive oil by-products have exhibited outstanding performance against CRC [17]. Therefore, the exploration of effective natural phytochemicals derived from olive tissue and olive oil by-products could aid in the prevention and treatment of colorectal cancer. Preliminary screening on pure molecules and/or natural extracts should be developed to evaluate the effect at a cellular level. Previous studies on the cytotoxic activity against human colorectal carcinoma, evaluated on HCT-116 cell line, reported a protective activity of several phenolic compounds as flavonoids, tannins, saponins, as well as of some terpenoids [18]. Several studies are available in the literature regarding the chemical characterization of olive fruits, olive pomace and olive leaves [19], while only a few studies have been reported concerning olive seeds, olive shells and olive branches [20,21]. However, most of the available studies were compiled with discontinuous investigation, considering different cultivars, growing areas, maturity stages and so on. Also, until now, the proposed methods have not evaluated the profile of phenolic compounds and triterpenoids in an overall way for all the possible by-products of the olive oil supply chain. Only few are part of in-depth studies involving a systematic approach on several olive tissues and by-products deriving from the same varieties for characterizing their phenolic and triterpenoid content. Further, as far as the authors are aware, no investigations have been reported on the effects of the phytocomplexes derived from a pool of olive tissue and olive oil by-products characterized in terms of phenolic and triterpene content on human colorectal carcinoma HCT-116 cell line.

In this study, three cultivars, namely Frantoio, Leccino, and Moraiolo, were selected to explore the maximal valorization potency of olive tissues and olive oil by-products. To reduce time-consumption, we developed a procedure for simultaneous measuring of both phenolic compounds and triterpenoids in all samples. The content of each detected compound and the total amount was determined for phenols and triterpenoids in olive leaf, branch, destoned fruit, destoned pomace, shell, seed, and EVOOs derived from the three cultivars. A preliminary screening of all the extracts from Frantoio cultivar was carried out on human colorectal cancer cell HCT-116 to assess the cytotoxicity of the phytocomplexes, comparing the results with those from some pure compounds.

## 2. Materials and Methods

### 2.1. Plant Materials

Samples were collected from an orchard on the hills close to Florence (Tuscany, Italy). They were tissues, by-products and virgin olive oils obtained from three cultivars (i.e., *Frantoio*, *Leccino* and *Moraiolo*) typical of Tuscany, but spread across the world. All samples were collected in the last decade of October 2019. Ten olive trees of each of the three cultivars were selected. Olive fruits, leaves and branches were collected from the selected plants around the entire canopy circumference. Extra virgin olive oils (EVOOs) were obtained by processing olives in a modern plant provided with a blade cutter crusher, a sealed vertical malaxer working under light vacuum conditions, a two-phase decanter (horizontal centrifuge) and a filter press for immediately filtering the obtained virgin olive oil. Finally, olive pomace were collected during the extraction of the corresponding EVOOs. Upon collection, samples arrived in the laboratory and were carefully manipulated as described in detail in Table 1. In this study, the terms fruit and pomace indicate that both of them were destoned.

### 2.2. Chemical Agents

All chemicals applied for analysis were of analytical grade. Formic acid, phosphoric acid, methanol and acetonitrile of HPLC grade were purchased from Sigma Aldrich (Steinheim, Germany) and Merck (Darmstadt, Germany), respectively. Deionized water was produced by the Milli-Q-system (Millipore SA, Molsheim, France). The standard compounds, syringic acid, pinoresinol, tyrosol, maslinic acid, oleanolic acid, ursolic acid and erythrodiol from Sigma Aldrich and esculetin, taxifolin, eriodictyol, luteolin-7-*O*-glucoside, verbascoside, nüzhenide, rutin and oleuropein were purchased from Extrasynthese Corporation (Genay, France), stock solutions of which were prepared in hydroalcoholic solution. MTS tetrazolium aqueous solution (the CellTiter 96^®^AQ_ueous_ One Solution Assay) was purchased by Promega Corportation (Madison, WI, USA).

### 2.3. Moisture Content

A moisture meter (model MB 23, Ohaus Co., Ltd., Nänikon Switzerland) was employed to measure the moisture content of samples after drying them at 105 °C for 15 min. The moisture levels in EVOOs, particularly in the filtered ones, are negligible based on preliminary reports [22]. The results from water contents for all samples can be found in Table S1 of Supporting Information.

### 2.4. Extraction Protocols

The extraction procedure was slightly modified from that of a previous manuscript [23]. Approximately 3.0 g of raw material powder of each sample (EVOO or 6 tissues/by-products) were mixed with EtOH:H_2_O 90:10 *v*/*v* in the solid-liquid ratio of 1:30. As Figure S1 shows (Supporting Information), they were extracted for 30 min at room temperature under ultrasound-assisted extraction with ultrasonic cleaner (DK Sonic Corporation, Shenzhen, China). Its parameters include model DK-300D, 40 KHz, ultrasonic power 120W, heating power 100 W and power supply in AC200-240V, 50Hz. Next, the extract solution was collected by filter under vacuum, and vacuum dried by rotation distill evaporator until the solid residue weight constant. Finally, they were re-suspended with water with the help of ultrasounds and transferred into falcon tube for lyophilization at −20 °C under vacuum. All extractions were performed in triplicate. All the test analytes were prepared 10.0 mg/mL in dimethyl sulfoxide (DMSO) or MeOH:H_2_O 80:20 *v*/*v*.

### 2.5. Simultanous Measurement of Phenolic Compounds and Triterpenoids by HPLC-DAD-MS

The previous methods for phenolic compounds [24,25] were modified to simultaneously determine phenolic compounds and triterpenoids in leaves, fruits, pomace, seeds, shells, branches and EVOO by HPLC-DAD applying one single analysis. Specifically, the HPLC instrumentation was a 1260 Infinity II LC System equipped with both Diode Array Detector (DAD) and Mass Spectrometry Detector (InfinityLab LC/MSD) with an API-electrospray interface (Agilent, Santa Clara, CA, USA). A Poroshell 120, EC-C18 (150 mm × 3.0 mm id, 2.7 µm particle size) column from Agilent Technologies, with a precolumn of the same phase was used with the temperature kept at 26 °C. Mobile phase was constituted by acetonitrile (A) and H_2_O with pH 3.2 by formic acid (B). Flow rate was 0.4 mL min^−1^ and the injection volume ranging from 5 to 10 µL. Detected wavelengths were set at 210, 240, 280 and 350 nm. Multistep linear gradient was exerted as follows: A started with 5% at 0.1 min, changed from 5% to 40% at 40 min, remaining at 40% until 45 min, then increased to 70% at 50 min, remaining at 70% until 60 min; increased to 100% at 65 min, remaining at 100% until 68 min, finally, it returned to 5% at 70 min.

Phenolic compounds were quantitated using calibration lines of tyrosol (λ = 280 nm, linearity range 0–1.21 µg; R^2^ = 0.9999), rutin (λ = 280 nm, 0–2.25 µg, R^2^ = 1.0000), luteolin-7-*O*-glucoside (λ = 280 nm, 0–2.81 µg, R^2^ = 0.9991), oleuropein (λ = 280 nm, 0–5.96 µg, R^2^ = 0.9972), nüzhenide (λ = 240 nm, 0–1.24 µg, R^2^ = 1.0000), verbascoside (λ = 280 nm, 0–1.96 µg, R^2^ = 0.9994) and pinoresinol (λ = 280 nm, 0–1.09 µg, R^2^ = 0.9999). Accordingly, individual phenolic compounds were showed as follows: tyrosol, hydroxytyrosol, glycosylated hydroxytyrosol and glycosylated tyrosol as mg_tyr_/kg; rutin as mg_rut_/kg; all flavonoids other than rutin as mg_lut_/kg; oleuropein, demethyloleuropein, ligstroside and their secoiridoid derivatives as mg_ole_/kg; nüzhenide, nüzhenide 11-methyl oleoside and their derivatives as mg_nuzh_/kg; cinnamic derivatives, verbascoside and related compounds as mg_verb_/kg; lignans as mg_pin_/kg. The total content of phenolic compounds has also been evaluated, including the area of all peaks in the chromatogram (both at 240 and 280 nm), for keeping into account all minor compounds; it was expressed as mg_ole_/kg according to our previous study [26]. Triterpenoid compounds were quantitated using calibration lines of maslinic acid (λ = 210 nm, linearity range 0–10 µg; R^2^ = 0.9979), oleanolic acid (λ = 210 nm, linearity range 0–12.5 µg; R^2^ = 0.9948), ursolic acid (λ = 210 nm, linearity range 0–10 µg; R^2^ = 0.9995) and erythrodiol (λ = 210 nm, linearity range 0–10 µg; R^2^ = 0.9957). Total triterpenoid levels were expressed as sum of all the detected triterpenoids. All triterpenoids were detected and quantitated at 210 nm; phenolic compounds were detected and quantitated at 280 nm, with the exception of nüzhenide, nüzhenide 11-methyl oleoside and their derivatives, which were detected and quantitated at 240 nm.

Phenolic compounds in the three EVOOs were measured according to the IOC official method [27]. Briefly, phenolic compounds were extracted by MeOH:H_2_O 80:20 *v*/*v* and instantly committed to analysis, which was executed by an HP1100 liquid chromatograph system provided with an auto-sampler and HP1100 DADs (Agilent Corporation, Santa Clara, CA, USA). A SphereClone ODS (2), 5 µm, 250 × 4.6 mm i.d. column was employed; the acid H_2_O (0.2% H_3_PO_4_)/acetonitrile/methanol gradient reported in the official method was used as elution with an injection volume of 20 μL at 280 nm. Their quantitation was implemented by the internal standard method, where syringic acid was acted as an internal standard concerning reference tyrosol. Thence, all the phenolic compounds such as secoiridoids, flavonoids, phenolic alcohols and acids were expressed as mg of tyrosol per kilogram of oil (mg_tyr_/kg_oil_).

### 2.6. HCT-116 Colon Cell Cytotoxicity Assessment with MTS Test

Cytotoxic activity assays were performed in 96 MW (6000 cells/well) after 72 h treatments. The medium volume for each well was 200 μL and each point was run in triplicate. At the end of the treatment, the media were removed, and cell monolayers were washed two times with 100 μL PBS. Subsequently, 100 μL/well of RPMI1640 (without phenol red and with 5% FBS) and 20 μL of ready-to-use MTS solution (CellTiter 96^®^ AQueous One Solution Cell Proliferation Assay, Promega) were added to each well and incubated in 5% CO_2_ humidified atmosphere at 37 °C until the color developed (about 30 min). The measurement of absorbance at 490 nm was performed in a multi-plate reader (VICTOR^3^ 1420 Mutilabel Counter, PerkinElmer Inc., Waltham, MA, USA) equipped with WALLAC software.

### 2.7. Data Analysis

All the trials were executed in triplicate and evaluated by Excel 2016 (Microsoft Inc., Redmond, Washington, DC, USA). One-way Analysis of Variance (ANOVA) was used for verifying the presence of significant differences among samples; when such a presence was confirmed, Fisher’s LSD test was applied to differentiate between mean values. Experiments from cancer colon cells were replicated 3 times and analyzed by means of GraphPad Prism (GraphPad Prism Software Inc., San Diego, CA, USA).

## 3. Results and Discussion

### 3.1. Phenolic Compounds in the Extracts from Different Products of Frantoio, Leccino and Moraiolo Cultivars

**Figure 1:**
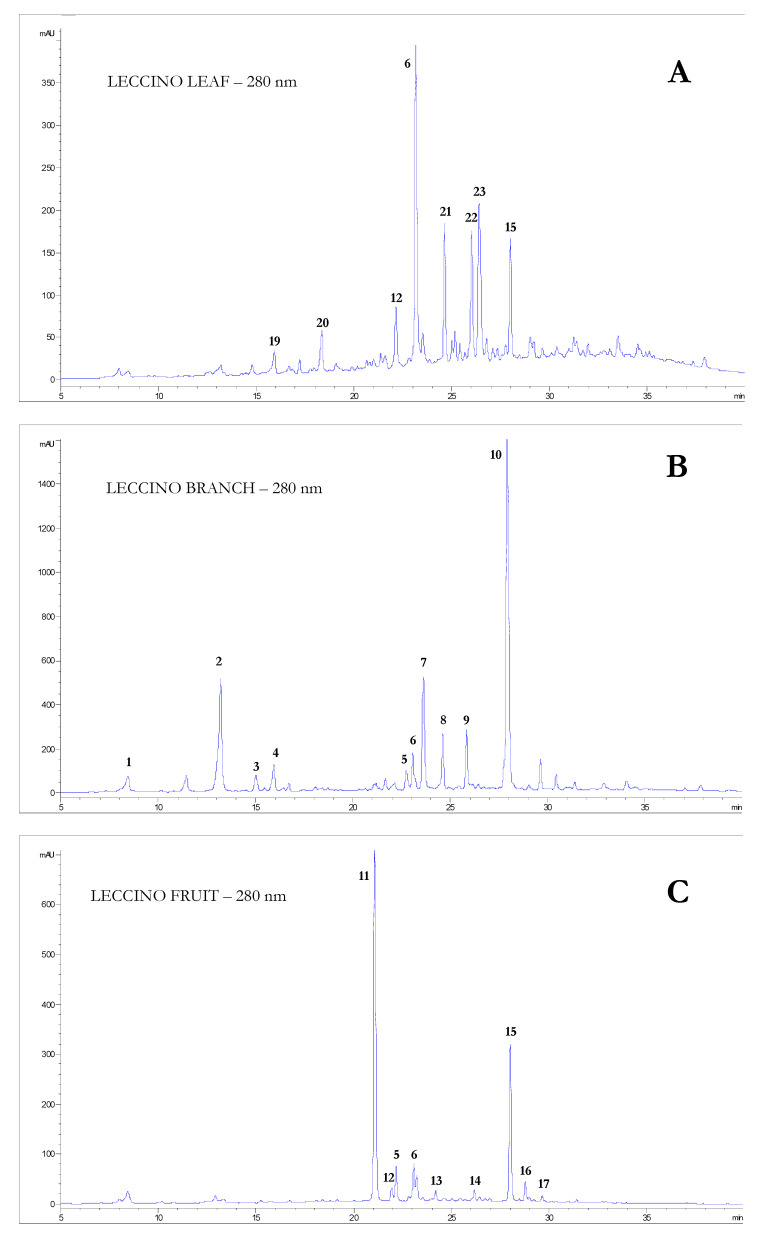

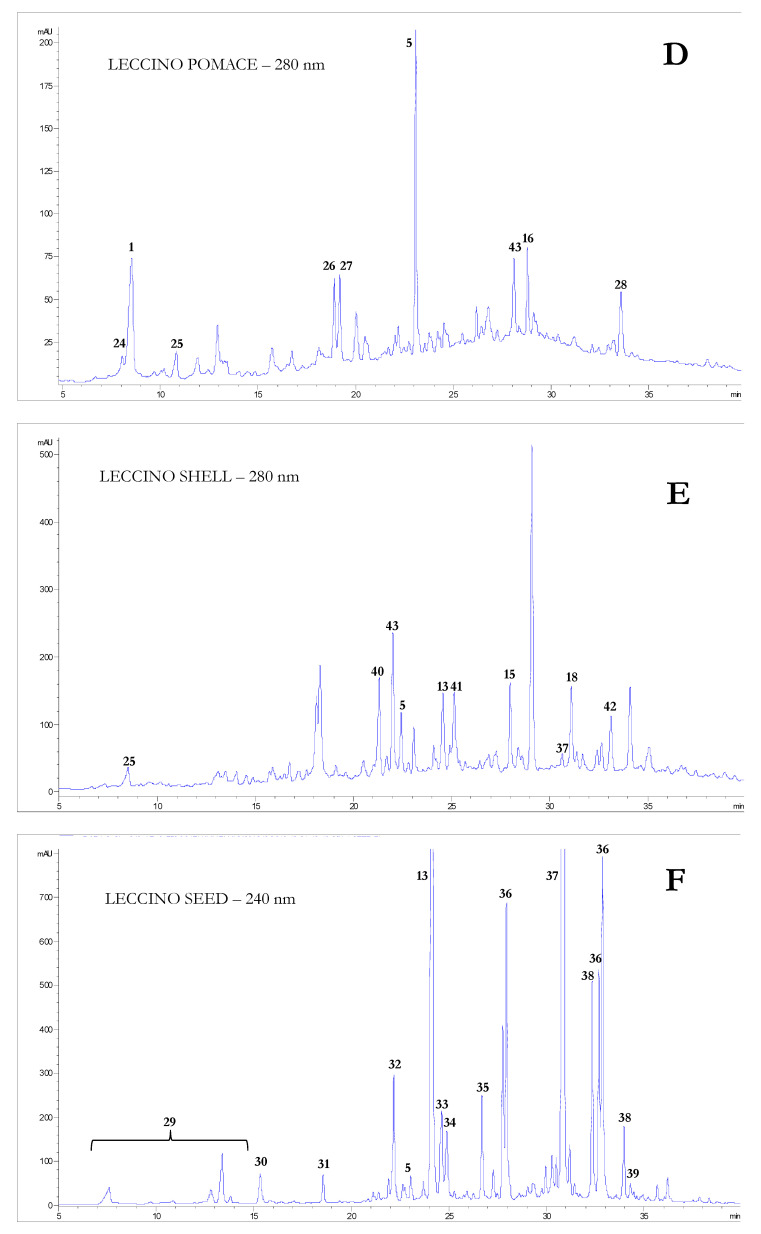
Chromatographic profiles at 280 nm of the phenolic compounds in Leccino cultivars: (**A**) Leaves, (**B**) Branches, (**C**) Fruits, (**D**) Pomace, (**E**) Shell and at 240 nm for (**F**) Seeds. 1, hydroxytyrosol; 2, taxifolin glucoside isomer; 3, esculetin; 4, eriodictyol-7-*O*-glucoside isomer; 5, verbascoside; 6, luteolin-7-*O*-glucoside; 7, taxifolin isomer; 8, oleuropein diglucoside (+comselogoside isobar); 9, 1-acetoxypinoresinol glucoside; 10, eriodictyol isomer; 11, demethyloleuropein; 12, rutin; 13, nüzhenide; 14, cafselogoside; 15, oleuropein; 16, comselogoside; 17, oleuropein aglycone; 18, ligstroside; 19, caffeic acid; 20, luteolin-3,7-diglucoside; 21, luteolin derivative; 22, apigenin glucuronide; 23, luteolin-4-*O*’-glucoside; 24, hydroxytyrosol glucoside; 25, tyrosol; 26, β-OH acteoside 1; 27, β-OH acteoside 2; 28, luteolin; 29, tyrosol derivatives; 30, oleoside 11-methyl ester; 31, oleoside 11-methyl ester isomer; 32, nüzhenide derivative; 33, bis(oleoside 11-methyl ester) glucoside; 34, salidroside oleoside; 35, nüzhenide isomer; 36, nüzhenide 11-methyl oleoside isomers 1-3; 37, nüzhenide 11-methyl oleoside; 38, nüzhenide di-(11-methyl oleoside) isomers 1-2; 39, ligstroside oleoside; 40, lariciresinol-sesquilignan + hydroxytyrosol acetate; 41, cinnamic derivative; 42, pinoresinol; 43, secoiridoid.

**Figure 2:**
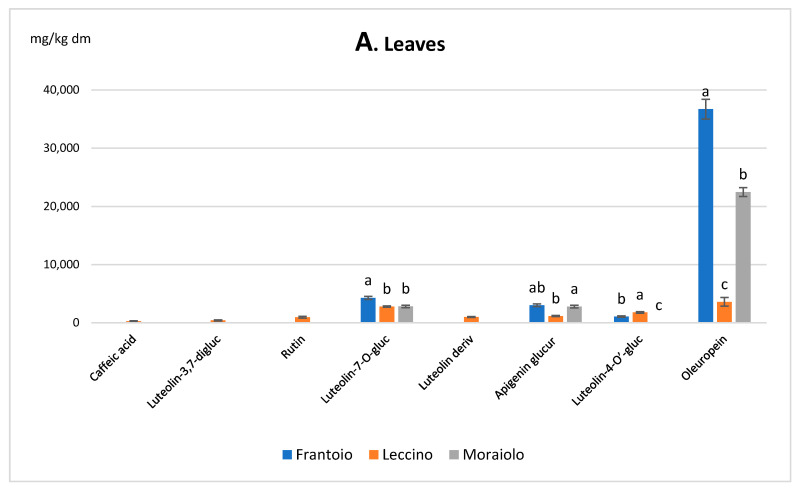

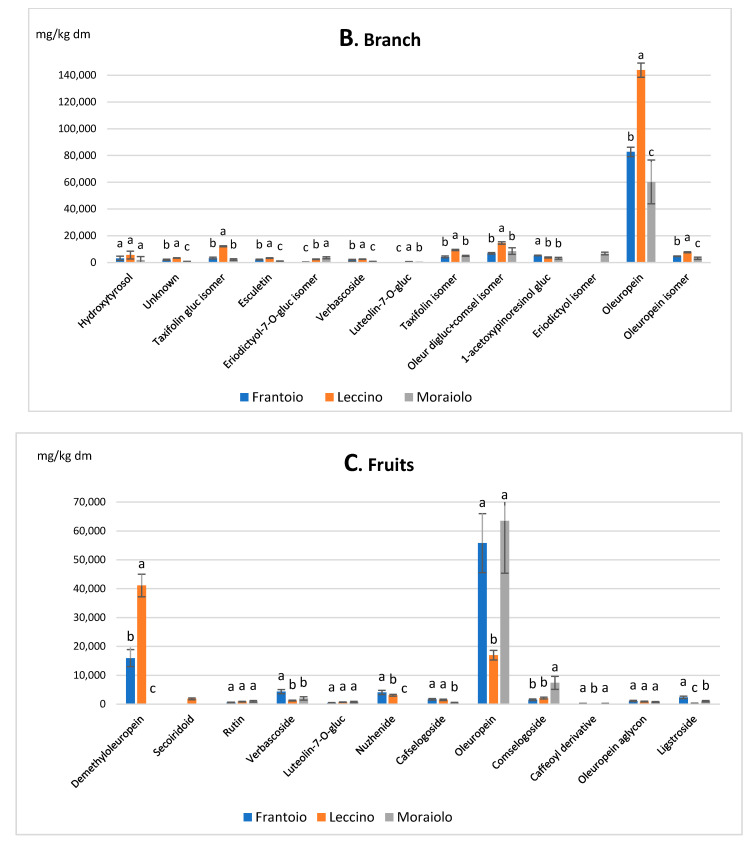

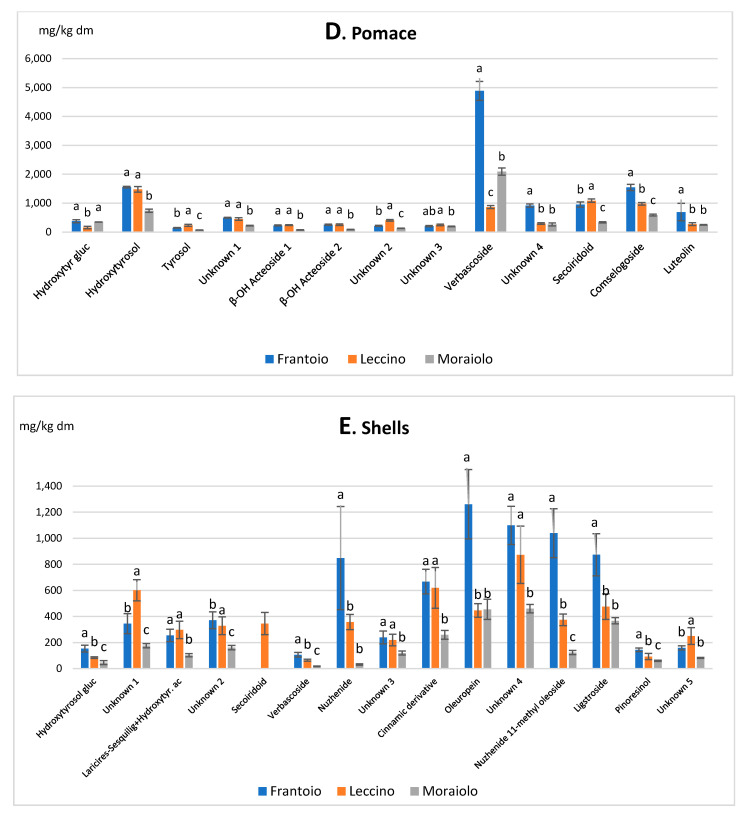

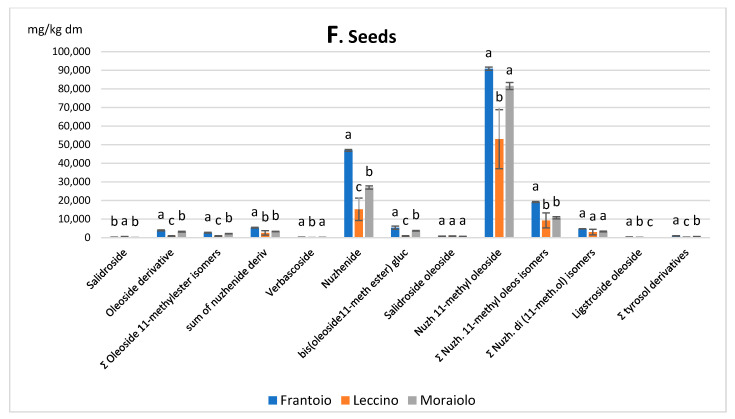
Distribution of phenolic compounds in the different extracts of the three varieties: (**A**) Leaves, (**B**) Branches, (**C**) Fruits; (**D**) Pomace, (**E**) Shell, (**F**) Seeds. For each molecule, different letters indicate different contents among the cultivar (the amount of each compounds ± SD is also reported in Table S3).

The characterization of olive tissues, virgin olive oil and by-products, and the applied treatment are summarized in Table 1. The chromatographic profiles in Figure 1 show the wide variety of phenolic compounds detected in the different olive tissues and virgin olive oil by-products samples, whereas Table S2 lists the MS fragmentation of individual phenolic compounds of the tested extracts. The histograms in Figure 2 aid in comparing the amount of each single phenol in leaves (A), branches (B), fruits (C), pomace (D), shells (E), and seeds (F) within the *Frantoio*, *Leccino*, and *Moraiolo* extracts. For each cultivar, a very similar distribution of phenol concentrations among the olive tissue samples is observed. Oleuropein was the main compound in leaves and branch for the three cultivars, as the similar results previously reported [28,29].

Concerning olive leaves, oleuropein showed the greatest level in *Frantoio*, reaching 36.71 g/kg and was over 10 and 1.63-fold higher than in *Leccino* and *Moraiolo* leaves, respectively. Also, luteolin-7-*O*-glucoside (4.29 g/kg) and apigenin derivative 2 (3.01 g/kg) showed the highest level in *Frantoio* leaves; the content of luteolin-7-*O*-glucoside in leaves of the other two cultivars was approximately 2.80 g/kg, while the apigenin derivative 2 was present in *Leccino* and *Moraiolo* leaves with a content of 1.16 g/kg and 2.78 g/kg, respectively. Luteolin-4-*O’*-glucoside was only present in the leaves of *Frantoio* (1.07 g/kg) and *Leccino* (1.78 g/kg). Interestingly, *Leccino* leaves showed the highest number of detected phenolic compounds, although their levels were not the highest. For this reason, this cultivar was chosen to represent the diversity of the phenolic profiles of the different samples (Figure 1). Low amounts of caffeic acid, luteolin-3,7-diglucoside, apigenin derivative 1 and rutin were observed in the leaves of *Leccino*, while these molecules were absent in the other two cultivars (Figure 2A and Table S3A).

As for the branch extracts, oleuropein showed very high concentrations, with the highest level in the *Leccino* samples (143.8 g/kg), approximately two-fold higher than that in *Frantoio* and *Moraiolo* samples. Also, the two co-eluting molecules, oleuropein diglucoside and comselogoside isobar (Figure 1), showed the greatest level (14.70 g/kg in total) in *Leccino* sample, almost two-fold greater than the other two cultivars. The flavanonols taxifolin and taxifolin glucoside isomers, and the coumarin derivative esculetin showed quite a higher content in *Leccino* branches than in those of the other two cultivars. Also, both hydroxytyrosol (5.65 g/kg) and verbascoside (2.52 g/kg) showed the highest level in *Leccino* branch. Overall, all the detected phenolic compounds in the branch of *Leccino* hold a greater concentration than those in *Frantoio* and *Moraiolo*, with the exception for 1-acetoxypinoresinol glucoside and eriodictyol-7-*O*-glucoside isomer. The results showed that *Frantoio* branches had the highest level of 1-acetoxypinoresinol glucoside (5.28 g/kg) and that the *Moraiolo* branches had the highest level of eriodictyol-7-*O*-glucoside isomer (3.54 g/kg). Esculetin may be considered as a marker compound for olive branch of at least these three cultivars (further studies will need to confirm this result also for other cultivars), in that it is not present in other olive tissues, according to the latest novelty search from the database of Scopus and Web of Knowledge. Eriodictyol isomer was only found in branches of *Moraiolo* and not in *Frantoio* and *Leccino* (Figure 2B and Table S3B).

For olive fruit, oleuropein level in *Frantoio* (55.79 g/kg) is appreciably lower than that in *Moraiolo* (63.54 g/kg), not less than that in *Leccino* (16.97 g/kg). An opposite behavior was highlighted for demethyloleuropein, obtained from the action of an endogenous esterase on oleuropein, which was present in the highest amount in the fruits of the *Leccino* cultivar (41.11 g/kg), and followed by *Frantoio* (15.96 g/kg), while it was not detected in the *Moraiolo* cultivar, as already reported in a previous study [26]. The cumulative sum levels of oleuropein and its derivatives (oleuropein, demethyloleuropein and oleuropein aglycon) gave the highest level (72.88 g/kg) in *Frantoio*, obviously higher than those reported in *Leccino* (58.98 g/kg) and *Moraiolo* (64.28 g/kg). Also, other secoiridoids as nüzhenide and ligstroside, and the phenylpropanoid derivative—namely, verbascoside—dominated in *Frantoio*, with greater values compared to *Moraiolo* and *Leccino*. Comselogoside was identified in all the three cultivars of olive fruits, and the highest level of 7.40 g/kg was presented in *Moraiolo*. As previously reported in other studies, comselogoside was present in olive fruits of *Frantoio* from Australia [29] and in the unripe olives of the studied Tuscan cultivars [30]. Besides, little amounts of rutin, luteolin-7-*O*-glucoside and caffeoyl derivative were observed in the three cultivars.

In olive pomace, as Figure 2 and Table S3 indicated, oleuropein was not found; however, hydroxytyrosol and its glucoside, as well as tyrosol, were detected in high concentrations [14]. This observation was opposite for olive fruit, which is possibly due to endogenous enzymes such as *β*-glycosidase acting for degradation of oleuropein after olive fruits rupture during olive oil extraction [31]. Verbascoside was the most abundant compound among the detected ones in olive pomace for the *Frantoio* and *Moraiolo* cultivars, reaching 4.88 g/kg and 2.09 g/kg, respectively. Likewise, comselogoside was found in olive pomace as well. Little amounts of the two *β*-OH acteoside isomers, which are hydroxylated derivatives of verbascoside, as well as of luteolin, were also identified for the three cultivars. Four unknown compounds were also identified, most likely indicating secoiridoids degradation, as previously shown in olive pomace [26].

Olive stones are made up of shells and seeds and are one of the most important and substantial biomasses, which contain high-value-added bioactive compounds in terms of phenols and triterpenoids. Overall, among the analyzed tissues and by-products, the shell samples were those with the lowest content of the identified phenolic compounds. As Figure 2E and Table S3E indicated, oleuropein, ligstroside, nüzhenide and nüzhenide11-methyl oleoside were the most abundant phenols in olive shell. Little amounts of hydroxytyrosol glucoside and acetate, verbascoside and the lignan pinoresinol were ascertained. From the viewpoint of plant physiology, olive fruit and shell being so adhesive, shown similar bioactive compounds. Five compounds detected in the olive shell of all the three cultivars are still unknown and almost all showed the highest content in *Frantoio*.

For olive seeds from Figure 2F and Table S3F, nüzhenide and its 11-methyl oleoside were by far the most concentrated phenols in the three cultivars, reaching the highest values (46.96 g/kg and 90.91 g/kg, respectively) in the *Frantoio* sample. The sum of nüzhenide and its derivatives reached 275.62 g/kg in the olive seed of *Frantoio*, and to the best of our knowledge this is the highest level reported till now in the literature. Other authors found that nüzhenide and nüzhenide 11-methyl oleoside were the dominant molecules in the respective seeds and reported for ‘*Lentisca*’ cultivar 12.2 g/kg of nüzhenide and 16.1 g/kg of nüzhenide 11-methyl oleoside expressed on fresh matter [21,32]. The sum of the eight tyrosol derivatives, including salidroside and salidroside oleoside, reached 2.05 g/kg in *Frantoio*. The sum of oleoside derivative, oleoside 11-methyl ester and its isobars achieved 6.69 g/kg. Little amounts of verbascoside and ligstroside oleoside were also found. Bis (oleoside 11-methyl ester) glucoside, a molecule never reported to date in the literature to the authors’ knowledge, is widely distributed in the three cultivars. All the detected compounds are more concentrated in *Frantoio* seeds.

EVOOs contain several phenols, mainly derived from oleuropein and ligstroside, which, during the milling process, are firstly transformed by endogenous *β*-glycosidase in their aglycon forms [33]. The loss of glucose enables the opening of the elenolic ring moiety giving chemical rearrangements resulting in the formation of new phenolic structures, with several tautomer forms (i.e., mono- and dialdehydic forms) which after the loss of carboxy-methyl group produce oleacin and oleocanthal [34,35]. Among EVOOs, *Frantoio* cultivar showed the highest total phenolic content (569.9 mg/kg, Table S4), 12.9% and 38% higher than *Leccino* and *Moraiolo*, respectively. Low amounts of free phenols as hydroxytyrosol, tyrosol, phenolic acids and flavonoids were observed, with values less than 6.4 mg/kg. The level of oleuropein derivatives (e.g., dialdehydic and aldehydic forms of decarboxymethyloleuropein aglycone and oleuropein aglycone) were high in each cultivar. Particularly, 240.4 mg/kg of total oleuropein derivatives in *Frantoio*, 267.6 mg/kg in *Leccino* and 219.1 mg/kg in *Moraiolo* were found. The total amounts of lignans as pinoresinol and 1-acetoxypinoresinol, were comparable to those of oleuropein for *Frantoio*, *Leccino* and *Moraiolo*, reaching 40.2 mg/kg, 18.8 mg/kg and 3.2 mg/kg, respectively. Likewise, EVOO from *Frantoio* showed much higher concentrations than the others (Table S4).

### 3.2. Triterpenoids Level in Each Olive Extract for the Three Varieties

Previous studies showed the presence of triterpenoids in olive oil, and also in leaves, fruit, and pomace [36], with maslinic, oleanolic, and ursolic acids, as well as erythrodiol, previously found in leaves and branches [7]. In our study, Figure S2 shows the HPLC profile of a mixture of commercial standards of the 4 triterpenoids, and the data in Table 2 (evaluated using the calibration curves reported in Table S5) indicates that the content of each of the four terpenoids varies greatly among the different samples. Maslinic and oleanolic acids were found in most of the extracts from the three varieties, particularly in fruit, leaves, pomace, and branches, but were absent in shells and seeds. Ursolic acid and erythrodiol were only detected in few tissues such as leaves and branches. All the four triterpenoids were found in EVOOs.

Among the cultivars, the amount of each terpenoid varies greatly. The highest concentration of maslinic acid was in fruit (5805 mg/kg) and branches (5916 mg/kg) of *Moraiolo*. Oleanolic acid showed the maximum amount in *Frantoio* leaves (17,036 mg/kg) and branches (6027 mg/kg). Analogously, ursolic acid was mainly present in leaves and branches, with the lowest concentration in *Frantoio* extracts. Overall, the content of erythrodiol was the lowest among the four terpenoids and it was undetected in the extracts from fruit, pomace, and shell of the three cultivars. The maximum number of total triterpenoids were in leaf and branches (from 14,134 mg/kg to 25,347 mg/kg), while the lower content per dry matter was in pomace (Table 2). None of the four terpenoids were detected in the shell extracts and only the *Leccino* seeds showed the presence of ursolic acid, while erythrodiol, maslinic and oleanolic acids were not detected in any seed extracts. All the four triterpenoids were found in EVOOs, with maslinic acid as the most abundant in *Leccino* (126.40 mg/kg) compared with the other cultivars. To summarize, the maximum number of total triterpenoids was detected in *Moraiolo* with 293.5 mg/kg (43% and 7% more than *Frantoio* and *Leccino*, respectively), the cultivar *Moraiolo* showed the highest concentration of oleanolic acid, and erythrodiol, whereas maslinic and ursolic acids were more abundant in *Leccino.*

### 3.3. Cytotoxicity Evaluation of HCT-116 Human Colorectal Cell for the Olive Extracts from Frantoio

**Figure 3:**
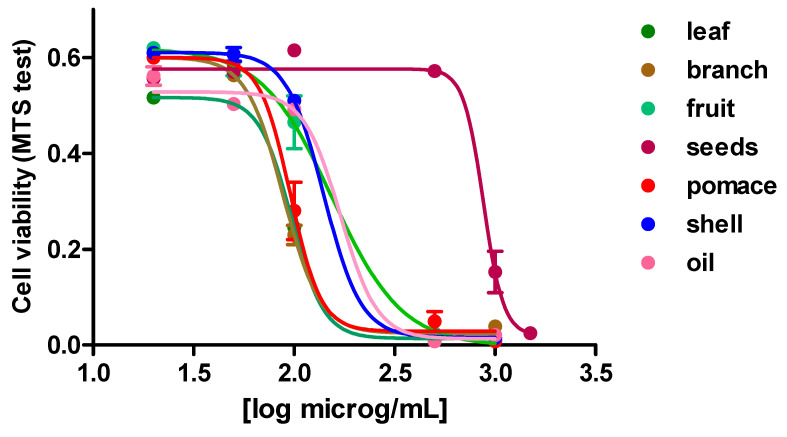
Dose-dependent cytotoxicity of the phenolic and triterpenoid extracts from *Frantoio* on HCT-116 human cancer cell line. Data are the mean ± SE of 3 independent experiments.

The cytotoxicity of the above-mentioned olive tissue and olive by-product extracts on colorectal cell HCT-116, was studied selecting the phytocomplex of the cultivar *Frantoio*, which was overall the richest one in phenols and triterpenoids. As Figure 3 shows, all the extracts exhibited a dose-dependent cytotoxic response on HCT-116 colorectal cells. The most active extracts in reducing colorectal cell viability were derived from branch, pomace, and leaf, with IC_50_ values of 88.25 μg/mL, 95.85 μg/mL and 97.06 μg/mL, respectively. The olive shell, olive fruit and olive oil extracts gave a weaker inhibition (IC_50_ values of 140.5 μg/mL, 154.3 μg/mL, and 170.0 μg/mL, respectively). The weakest cytotoxic effect on colorectal cells was shown by the olive seed extract with a high IC_50_ value (875.5 μg/mL). Single phenolic compounds and triterpenoids have been previously reported to inhibit proliferation in colorectal cancer cell [37,38]. However, the different chemical profiles of each extract can exert a strong influence on the toxicity of the sample. As mentioned above, the olive seed extract was the richest in glycosylated secoiridoids analogous of nüzhenide, with a very different phenolic profile compared with the other samples. The IC_50_ of leaf and branch extracts were similar, and these two extracts were indeed characterized by the highest terpenoid content and a high concentration of oleuropein.

To evaluate the role of the phenolic compounds and triterpenoid, and keeping in mind their presence in the different samples, some representative pure compounds were also included in the present study for determining their cytotoxic activity in HCT-116 cells. Among the phenolic compounds, oleuropein can be cited as the most abundant in leaf, branch, fruit, and shell extract, hydroxytyrosol in pomace, and nüzhenide in seeds. For triterpenoids, maslinic acid and oleanolic acid were well represented in olive fruit, leaf, pomace, and branch, while were absent in seeds from *Frantoio*. Among the tested terpenoids, maslinic acid showed one of the lowest IC_50_ values along with ursolic acid (Table 3); noteworthy, this latter triterpenoid was detected only in branches but not in the other samples. Oleanolic acid could not be further evaluated due to its scarce solubility in water.

Phenols have been reported to inhibit growth and to induce apoptosis of HCT-116 cells via reactive oxygen species (ROS) generation triggered by the activation of mitochondria-mediated intrinsic pathways and the blockage of NF-κB signaling pathway downstream of ROS generation [39]. However, natural phenols can bring about chemoprevention, not only interfering with different kinds of oxidative stress, but also interacting with all the main signaling pathways in cancer cells [40,41]. The *o*-catechol structure and the number of hydroxyl groups appeared to correlate with cytotoxic activity. For oleuropein, it has been proposed that the iridoid terpene moiety cooperates with the catechol structure in inhibiting HCT-116 cell proliferation [42]. However, our results show almost the same value for the IC_50_ of hydroxytyrosol and oleuropein, suggesting the *o*-catechol group present in the two molecules has the main effect.

Triterpenoids can induce apoptosis acting on multiple targets, for example on mitochondria by attacking the permeability transition pore complex, by restraining IAP proteins, or by inhibiting anti-apoptotic Bcl-2 proteins [43]. Nevertheless, the mitochondrial apoptotic pathway, activated through the mitochondrial release of cytochrome C into the cytosol, appears to be the main target of triterpenoids in colorectal cancer cells [44].

## 4. Conclusions

This study reported the complete profile of phenols and triterpenoids in various olive products extracts obtained from three Italian cultivars, also selected because they are widely distributed around the world. A simultaneous analytical determination was developed to simultaneously determine phenolic and triterpenoid compounds. With this approach, it was possible to verify that oleuropein is dominant in leaves, branches, and fruits, while verbascoside and hydroxytyrosol are the main phenolic compounds in olive pomace. The olive shell results in the lowest source of total phenols. As for the triterpenoids, olive leaves and branches contain maslinic acid, oleanolic acid, ursolic acid and erythrodiol, while only maslinic and oleanolic acids have been detected in the fruit and olive pomace. Ursolic acid and erythrodiol were only present in the seeds of Leccino, while all the other seeds were free from triterpenoids.

The study on cytotoxicity towards a colorectal cancer cell line, determined by working with extracts from the Frantoio cultivar, showed a dose-dependent relationship. Specifically, the most effective phytocomplex was obtained from the branch, followed by pomace and leaf extracts. Overall, the study allowed collecting chemical profiles of the dry extracts obtained from different tissues and by-products of *Olea europaea* L., and to evaluate their toxicity in a human colorectal cell line model, helping to hypothesize potential future uses of these products.

## Figures and Tables

**Table 1:** Description of the selected samples from the three varieties.

Oil and By-Products	Frantoio	Leccino	Moraiolo	Treatment Ways
Extra virgin olive oil	500 mL, light green	500 mL, yellowish	500 mL, light green	Directly obtained from olive mill for use
Olive leaf	6~7 × 1.4~1.5 cm	6~7 × 1.4~1.5 cm	5~6 × 1.4~1.5 cm	Naturally dried indoor at R.T. for 5 days, and pulverization
Olive pomace (none stone)	Gray-yellow powder after being lyophilized	Gray-yellow powder after being lyophilized	Gray-yellow powder after being lyophilized	Lyophilized for 2 days and then remove stone and pulverization
Olive fruit (none stone)	1.7~1.8 cm × 1.3~1.4 cm green & dark	1.8~1.9 × 1.3~1.4 cm mostly dark	1.7~1.8 × 1.2~1.3 cm green & dark	Lyophilized for 2 days and then destoned with manual handing and pulverization
Olive shell (none seed)	1.1~1.2 × 0.6~0.7 cm	1.3~1.5 × 0.65~0.7 cm	1.15~1.2 × 0.65~0.7 cm	Naturally dried indoor at R.T. for 5 days, and then separate seed cautiously with a hammer and pulverization
Olive seed	0.9~1.0 × 0.4~0.45 cm	1.0~1.1 × 0.4~0.5 cm	0.85~0.95 × 0.35~0.4 cm	The intact seed was kept in darkness and naturally dried for 3 days and pulverization
Olive branch	45~50 cm cut from the top with cut diameter: 3~5 mm containing 0.3~0.4 mm thickness of the peel	45~50 cm cut from the top with cut diameter: 3~5 mm containing 0.3~0.4 mm thickness of the peel	45~50 cm cut from the top with cut diameter: 3~5 mm containing 0.3~0.4 mm thickness of the peel	Naturally dried indoor at R.T. for 5 days, and pulverization

Note: Randomly select the 100 samples to measure their sizes. Leaf size of the width means the maximal level in an ellipse shape. The above data in Table 1 about olive byproducts were expressed as length × width. Also, R.T. means room temperature. All of these samples, except for extra virgin olive oil, were pulverized about 0.45 mm by a grinder (IKA-Werke Corporation, M20, Staufen, Germany) and mesher.

**Table 2:** Distribution of maslinic acid, oleanolic acid, ursolic acid and erythrodiol in all the extracts from the three Tuscan varieties determined by HPLC-DAD. For each molecule, different letters indicate different contents among cultivars.

		mg/kg						
Triterpenoid. (rt, min)	cv	Fruits	Leaves	Pomace	Branches	Shell	Seeds	Oil
Maslinic acid (49.5)	Frantoio	2277 c	2434 b	1319 b	1745 a	nd	nd	82.55 b
	Leccino	4003 b	2583 b	3442 a	3685 a	nd	nd	126.40 a
	Moraiolo	5805 a	4534 a	551 c	5916 a	nd	nd	36.97 c
Oleanolic acid (53.2)	Frantoio	1142 b	17036 a	783 a	6027 b	nd	nd	25.75 a
	Leccino	1925 a	11880 b	940 a	10104 a	nd	nd	37.70 a
	Moraiolo	2265 a	13121 b	367 b	11374 a	nd	nd	40.20 a
Ursolic acid (53.3)	Frantoio	nd	nd	nd	5386 a	nd	nd	46.01 b
	Leccino	nd	5556 a	738	5321 a	nd	7338	92.63 a
	Moraiolo	nd	6334 a	nd	7605 a	nd	nd	46.80 b
Erythrodiol (55.4)	Frantoio	nd	3219 a	nd	976 b	nd	nd	51.00 b
	Leccino	nd	nd	nd	2148 a	nd	2717	16.97 c
	Moraiolo	nd	1358 b	nd	nd	nd	nd	169.53 a
Total Triterpenoids level	Frantoio	3419 c	22689 b	2102 b	14134 b	nd	nd	205.3 b
	Leccino	5928 b	20019 c	5120 a	21258 a	nd	10055	273.7 a
	Moraiolo	8115 a	25347 a	918 c	24895 a	nd	nd	293.5 a

**Table 3:** (**A**) IC_50_ values of the phenolic and triterpenoid extracts from *Frantoio* cv; (**B**) IC_50_ values of some representative pure phenols and triterpenoids.

A	Leaf	Branch	Fruit	Seeds	Pomace	Shell	Oil
IC50 (µg/mL)	97.06	88.25	154.3	875.5	95.85	140.5	170.0
**B**	**OH-Tyrosol**	**Verbascoside**		**Clorogenic acid**		**Caffeic acid**	**Oleuropein**
IC50 (µM)	66	79		207		145	61
	Rutin	Luteolin 7-*O*-glucoside		Quercetin		Luteolin	Taxifolin
IC50 (µM)	940	58		38		89	200
	Maslinic acid	Ursolic Acid		Erythrodiol			
IC50 (µM)	41	24		69			

## Data Availability

Data is contained within the article or supplementary material.

## Supplementary Materials

The following are available online at https://www.mdpi.com/article/10.3390/foods10112823/s1, Table S1: Dryness yield of the different extracts from the three Tuscan varieties; Table S2. MS fragmentation of individual phenolic compounds of the tested extracts; Table S3: Distribution of phenolic compounds in the different extracts (tissues): (A) Leaves, (B) Branches (C) Fruits; (D); Pomace (E) Shell; (F) Seeds; Table S4: Phenolic content in EVOOs; Table S5: Data concerning the calibration curves for the 5 external standards used for the quantitation of phenols and triterpenoids; Figure S1. The graphic scheme of this work; Figure S2. HPLC profile of triterpenoids for mixture standards: A, maslinic acid; B, oleanolic acid; C, ursolic acid; D, erythrodiol.

## Abbreviations

EVOOs, Extra Virgin Olive Oils; IOC, International Olive Council; HPLC-DAD-MS, High Performance Liquid Chromatograph Equipped with Diode Array Detector and Mass Spectrometer; DMSO, Dimethyl Sulfoxide; IC50, Half Maximal Inhibitory Concentration; CRC, Colorectal Cancer; MTS, 3-(4,5-Dimethylthiazol-2-yl)-5-(3-Carboxymethoxyphenyl)-2-(4-Sulfo-phenyl)-2H-Tetrazolium, Inner Salt; ROS, Reactive Oxygen Species; NF-κB, Nuclear Factor Kappa-B; IAP, Inhibitor of Apoptosis Protein; Bcl-2, B-Cell Lymphoma-2.
