# Supplementary material for: Phenolic Compounds and Triterpenes in Different Olive Tissues and Olive Oil By-Products, and Cytotoxicity on Human Colorectal Cancer Cells: The Case of Frantoio, Moraiolo and Leccino Cultivars (Olea europaea L.)
Source: Foods. 2021 Nov 16;10(11):2823. doi: 10.3390/foods10112823 (PMC8618932; doi:10.3390/foods10112823)

Supporting information file of the manuscript:

Article

Phenolic compounds and Triterpenes in Different Olive Tissues and Olive Oil By-Products, and Cytotoxicity on Human Colorectal Cancer Cells: the Case of Frantoio, Moraiolo and Leccino Cultivars (*Olea europaea* L.)

Pujun Xie [1,2,3,4,5], Lorenzo Cecchi [6], Maria Bellumori [6], Diletta Balli [6], Lisa Giovannelli [7], Lixin Huang [1,2,3,4,5] and Nadia Mulinacci [6,*]

[1] Institute of Chemical Industry of Forest Products, CAF. Nanjing 210042, China.
[2] National Engineering Laboratory for Biomass Chemical Utilization. Nanjing 210042, China.
[3] Key and Open Laboratory on Forest Chemical Engineering, SFA. Nanjing 210042, China.
[4] Key Laboratory of Biomass Energy and Material, Nanjing 210042, China; pujunxie@caf.ac.cn (P.X.); l_x_huang@163.com (L.H.)
[5] Co-Innovation Center of Efficient Processing and Utilization of Forest Resources, Nanjing Forestry University, Nanjing 210037, China
[6] University of Florence, Department of NEUROFARBA, Nutraceutical and Pharmaceutical Section, Via Ugo Schiff 6, Sesto Fiorentino, 50019 Florence, Italy; lo.cecchi@unifi.it (L.C.); maria.bellumori@unifi.it (M.B.); diletta.balli@unifi.it (D.B.)
[7] University of Florence, Department of NEUROFARBA, Pharmacology and Toxicology Section, Viale Pieraccini 6, 50139 Florence, Italy; lisa.giovannelli@unifi.it
* Correspondence: nadia.mulinacci@unifi.it; Tel.: +39-0554573773

Table S1. Dryness yield of the different extracts from the three Tuscan varieties.

**Samples as raw materials (g)**	***Frantoio***		***Leccino***		***Moraiolo***	
	**g dryness/g sample**	**SD**	**g dryness/g sample**	**SD**	**g dryness/g sample**	**SD**
Olive leaf	0.266	0.001	0.243	0.019	0.242	0.052
Olive branch	0.243	0.019	0.177	0.038	0.100	0.033
Olive pomace	0.343	0.019	0.398	0.002	0.439	0.019
Olive seed	0.244	0.012	0.211	0.050	0.232	0.004
Olive shell	0.042	0.007	0.027	0.005	0.024	0.007
Olive fruit	0.352	0.084	0.472	0.094	0.438	0.042
Olive oil (EVOO)	0.033	0.003	0.033	0.002	0.032	0.001

Note: water containing in the raw material is not included.

Table S2. MS fragmentation of individual phenolic compounds of the tested extracts

Compounds	λ_{max} (nm)	mw	Major ESI peaks
1-acetoxypinoresinol glucoside	280	578	577, 415
bis(oleoside 11-methyl ester) glucoside	235	952	951, 789, 403
caffeic acid	296, 325	180	179
cafselogoside	295, 328	552	551, 507, 389, 162
comselogoside	315	536	535, 491, 389, 345, 145
demethyloleuropein	280	526	525, 241
eriodictyol isomer	290	288	575, 287
eriodictyol-7-O-glucoside isomer	285, 336	450	449, 287
esculetin	268, 295, 343	178	177, 355
hydroxytyrosol	280	154	153
hydroxytyrosol glucoside	278	316	315, 153
hydroxytyrosol acetate	-	196	195
lariciresinol-sesquilignan	-	556	555
ligstroside	275	524	523
ligstroside oleoside	240	910	909, 523, 361
luteolin	348	286	285
luteolin derivative	266, 338	448	895, 447
luteolin-3,7-diglucoside	268, 336	610	609, 447, 285
luteolin-4-O'-glucoside	268, 336	448	895, 447
luteolin-7-O-glucoside	253, 344	448	895, 447, 285
nüzhenide	225, 280	686	685, 523, 223
nüzhenide 11-methyl oleoside	240	1072	1071, 909, 685, 523
nüzhenide 11-methyl oleoside isomers 1-3	240	1072	1071, 909, 685, 523, 299
nüzhenide derivative	230, 280	848	847, 685
nüzhenide di-(11-methyl oleoside) isomers 1-2	240	1458	1457
nüzhenide isomer	225, 280	686	685, 523, 453
oleoside 11-methyl ester	240	404	807, 403, 223, 179
oleoside 11-methyl ester isomer	240	404	807, 403, 223, 179
oleoside derivative	240	-	1149, 969, 951, 789, 565, 403, 223
oleuropein	280	540	539, 377, 307, 223
oleuropein aglycone	280	378	377, 307, 275
oleuropein diglucoside	280	702	701, 539
pinoresinol	280	358	357
rutin	254, 352	610	609, 301, 300
Salidroside	275	300	599, 299, 137
salidroside oleoside	235, 280	686	685, 523, 299
taxifolin glucoside isomer	285, 330	466	465, 303
taxifolin isomer	288, 330	304	303
tyrosol	276	138	137
verbascoside	285, 330	624	623, 461, 161
β-OH acteoside 1	285, 330	640	639, 621, 179, 161
β-OH acteoside 2	285, 330	640	639, 621, 179, 161

Table S3. Distribution of phenolic compounds in the different extracts (tissues): A) Leaves, B) Branches C) Fruits; D); Pomace E) Shell; F) Seeds.

A) Leaves

mg/kg dm	**Frantoio**	**Leccino**	**Moraiolo**
Caffeic acid	nd	286 ± 73	nd
Luteolin-3,7-diglucoside	nd	413 ± 83	nd
Rutin	nd	960 ± 166	nd
Luteolin-7-*O*-glucoside	4289 ± 263	2807 ± 113	2823 ± 208
Apigenin derivative 1	nd	997 ± 88	nd
Apigenin derivative 2	3014 ± 236	1164 ± 94	2779 ± 229
Luteolin-4-*O*’-glucoside	1066 ± 119	1782 ± 124	nd
Oleuropein	36710 ± 1708	3600 ± 744	22471 ± 766

B) Branch

mg/kg dm	**Frantoio**	**Leccino**	**Moraiolo**
Hydroxytyrosol	3216 ± 1511	5647 ± 2934	2010 ± 2438
Unknown	2142 ± 472	3449 ± 166	734 ± 171
Taxifolin glucoside isomer	3364 ± 713	12247 ± 376	2257 ± 653
Esculetin	2286 ± 333	3352 ± 196	1021 ± 222
Eriodictyol-7-*O*-glucoside isomer	240 ± 247	2466 ± 234	3540 ± 750
Verbascoside	1786 ± 674	2519 ± 120	795 ± 46
Luteolin-7-*O*-glucoside	nd	647 ± 39	181 ± 27
Taxifolin isomer	4197 ± 672	9416 ± 423	5017 ± 344
Oleuropein diglucoside + comselogoside isobar	6846 ± 607	14699 ± 915	8600 ± 2435
1-acetoxypinoresinol glucoside	5284 ± 251	3820 ± 316	3217 ± 697
Eriodictyol isomer	nd	nd	6786 ± 930
Oleuropein	82720 ± 3533	143794 ± 5293	60232 ± 16382
Oleuropein isobar	4739 ± 182	7730 ± 376	3274 ± 824

C) Fruit

mg/kg dm	**Frantoio**	**Leccino**	**Moraiolo**
Demethyloleuropein	15963 ± 2947	41111 ± 3887	nd
Secoiridoid	nd	1855 ± 323	nd
Rutin	605 ± 91	876 ± 118	970 ± 271
Verbascoside	4343 ± 709	1243 ± 150	1980 ± 587
Luteolin-7-*O*-glucoside	403 ± 133	694 ± 90	740 ± 228
Nüzhenide	4072 ± 721	3089 ± 322	nd
Cafselogoside	1689 ± 287	1497 ± 262	479 ± 135
Oleuropein	55788 ± 10231	16969 ± 1681	63539 ± 18161
Comselogoside	1534 ± 274	2113 ± 316	7401 ± 2243
Caffeoyl derivative	218 ± 44	nd	201 ± 50
Oleuropein aglycone	1129 ± 204	902 ± 152	744 ± 178
Ligstroside	2346 ± 413	313 ± 25	996 ± 262

D) Pomace

mg/kg dm	**Frantoio**	**Leccino**	**Moraiolo**
Hydroxytyrosol glucoside	378 ± 52	156 ± 43	348 ± 6
Hydroxytyrosol	1554 ± 31	1475 ± 96	738 ± 54
Tyrosol	144 ± 10	233 ± 36	71 ± 3
Unknown 1	493 ± 14	450 ± 47	225 ± 12
β-OH Acteoside 1	229 ± 15	238 ± 13	76 ± 6
β-OH Acteoside 2	255 ± 19	254 ± 29	87 ± 6
Unknown 2	214 ± 17	404 ± 27	130 ± 8
Unknown 3	206 ± 22	248 ± 29	198 ± 10
Verbascoside	4883 ± 329	865 ± 50	2093 ± 122
Unknown 4	918 ± 55	296 ± 25	265 ± 53
Secoiridoid	950 ± 89	1086 ± 59	338 ± 20
Comsecologoside	1546 ± 108	978 ± 44	589 ± 33
Luteolin	690 ± 301	273 ± 50	249 ± 11

E) Shell

mg/kg dm	**Frantoio**	**Leccino**	**Moraiolo**
Hydroxytyrosol glucoside	154 ± 26	85 ± 6	46 ± 15
Unknown 1	345 ± 76	601 ± 82	176 ± 16
Lariciresinol-sesquilignan + Hydroxytyrosol acetate	255 ± 47	297 ± 67	103 ± 12
Unknown 2	371 ± 64	329 ± 68	161 ± 17
Secoiridoid	nd	346 ± 84	nd
Verbascoside	107 ± 18	65 ± 8	19 ± 2
Nüzhenide	848 ± 396	358 ± 59	32 ± 5
Unknown 3	240 ± 49	220 ± 45	120 ± 15
Cinnamic derivative	668 ± 95	620 ± 156	259 ± 34
Oleuropein	1261 ± 266	446 ± 53	454 ± 78
Unknown 4	1099 ± 146	873 ± 219	460 ± 33
Nüzhenide 11-methyl oleoside	1039 ± 188	374 ± 44	124 ± 16
Ligstroside	873 ± 162	475 ± 98	367 ± 24
Pinoresinol	144 ± 14	93 ± 24	60 ± 5
Unknown 5	159 ± 16	250 ± 65	82 ± 4

F) Seeds:

mg/kg dm	**Frantoio**	**Leccino**	**Moraiolo**
Elenolic acid diglucoside	nd	nd	nd
Salidroside	270 ± 32	501 ± 165	140 ± 15
Oleoside derivative	3968 ± 288	748 ± 328	3242 ± 171
Oleoside 11-methyl ester	1473 ± 152	514 ± 181	1544 ± 71
Oleoside 11-methyl ester isobar	1246 ± 29	313 ± 132	581 ± 37
Nüzhenide derivative	2907 ±68	1511 ± 693	2445 ± 28
Verbascoside	258 ± 41	68 ± 25	237 ± 31
Nüzhenide	46963 ± 495	15305 ± 6042	26965 ± 849
Bis(oleoside 11-methyl ester) glucoside	5413 ± 894	729 ± 386	3725 ± 155
Salidroside oleoside	789 ± 138	705 ± 251	769 ± 10
Nüzhenide isobaric	2525 ± 133	1111 ± 464	930 ± 87
Nüzhenide 11-methyl oleoside is 1	6447 ± 44	3096 ± 1279	3588 ± 112
Nüzhenide 11-methyl oleoside	90909 ± 868	52977 ± 15895	81584 ± 1895
Nüzhenide di (11-methyl oleoside) is 1	3484 ± 34	2193 ± 1067	2962 ± 225
Nüzhenide 11-methyl oleoside is 2	6595 ± 92	2381 ± 1062	2732 ± 224
Nüzhenide 11-methyl oleoside is 3	6128 ± 127	3830 ± 1687	4441 ± 258
Nüzhenide di (11-methyl oleoside) is 2	1275 ± 21	833 ± 397	404 ± 12
Ligstroside oleoside	347± 71	196 ± 64	60 ± 7
Tyrosol derivative 1	115 ± 13	nd	70 ± 7
Tyrosol derivative 2	74 ± 10	nd	145 ± 9
Tyrosol derivative 3	153 ± 9	nd	27 ± 6
Tyrosol derivative 4	304 ± 17	110 ± 40	177 ± 5
Tyrosol derivative 5	188 ± 10	123 ± 43	122 ± 8
Tyrosol derivative 6	160 ± 9	37 ± 16	96 ± 6

Table S4. Phenolic content in EVOOs

Phenolic compounds (mg/kg)	Frantoio	Leccino	Moraiolo
Hydroxytyrosol	2.5 ± 0.2	2.1 ± 0.2	1.4 ± 0.1
Tyrosol	4.8 ± 0.2	2.6 ± 0.1	1.9 ± 0.1
Vanillic + caffeic acids	1.3 ± 0.0	1.2 ± 0.0	1.1 ± 0.0
Vanillin	2.9 ± 0.2	4.9 ± 0.3	2.2 ± 0.1
p-coumaric acid	1.1 ± 0.1	0.6 ± 0.1	0.7 ± 0.1
Hydroxytyrosol acetate	1.1 ± 0.1	2.9 ± 0.3	1.3 ± 0.1
Ferulic acid	4.3 ± 0.8	0.4 ± 0.1	0.6 ± 0.1
o-coumaric acid	1.2 ± 0.2	0.8 ± 0.2	0.6 ± 0.1
Dialdehydic form of decarboxymethyloleuropein aglycone oxidized	18.6 ± 1.6	19.6 ± 1.6	12.0 ± 1.0
Dialdehydic form of decarboxymethyloleuropein aglycone	103.5 ± 1.1	177.1 ± 1.9	77.4 ± 0.8
Oleuropein	36.9 ± 1.3	19.1 ± 0.7	26.9 ± 1.0
Dialdehydic form of oleuropein aglycone	16.3 ± 1.0	9.9 ± 0.6	12.3 ± 0.8
Dialdehydic form of decarboxymethylligstroside aglycone oxidized	91.8 ± 4.5	33.4 ± 1.6	49.5 ± 2.4
Dialdehydic form of decarboxymethylligstroside aglycone	77.6 ± 1.7	115.0 ± 2.5	39.6 ± 0.9
Pinoresinol + 1-acetoxy-pinoresinol	40.2 ± 1.0	18.8 ± 0.5	23.2 ± 0.6
Cinnamic acid	3.3 ± 0.5	2.2 ± 0.3	1.2 ± 0.2
Dialdehydic form of ligstroside aglycone	3.7 ± 0.4	3.5 ± 0.4	3.6 ± 0.4
Aldehydic and hydroxylic form of oleuropein aglycone oxidized	28.1 ± 1.3	16.7 ± 0.7	30.8 ± 1.4
Luteolin	6.4 ± 0.9	1.2 ± 0.2	1.6 ± 0.2
Aldehydic and hydroxylic form of oleuropein aglycone	73.9 ± 1.1	44.3 ± 0.6	86.6 ± 1.2
Aldehydic and hydroxylic form of ligstroside aglycone oxidized	23.9 ± 2.4	13.1 ± 1.3	13.6 ± 1.4
Apigenin	4.1 ± 0.4	3.6 ± 0.4	2.1 ± 0.2
Methyl luteolin	16.0 ± 1.3	9.6 ± 0.8	18.5 ± 1.5
Aldehydic and hydroxylic form of ligstroside aglycone	6.6 ± 0.2	2.1 ± 0.1	4.3 ± 0.2
Total phenolic compounds	569.9 ± 10.0	504.8 ± 8.9	413.0 ± 7.3

Table S5. Data concerning the calibration curves for the 5 external standards used for the quantitation of phenols and triterpenoids.

**Number**	**Standards**	**Wavelengths (nm)**	**Different solutions (μM)**	**Linear equitation**	**R^2**
1	hydroxytyrosol	280	800, 400, 160, 100, 50, 5	$Y = 0.783\ X + 6.684$	0.965
2	oleuropein	280	400, 200, 50	$Y = 0.845\ X + 12.183$	0.956
3	maslinic acid	210	100, 10, 1	$Y = 0.784\ X + 21.535$	0.999
4	oleanolic acid	210	20, 10, 1	$Y = 0.530\ X + 3.979$	0.837
5	nüzhenide	240	800, 400, 200, 50	$Y = 0.0144X + 12.817$	0.900

Note: *Y* indicates the HPLC peak at 280, 210 and 240 nm, and *X* indicates the molar concentrations for these standards.

Figure S1. The graphic scheme of this work

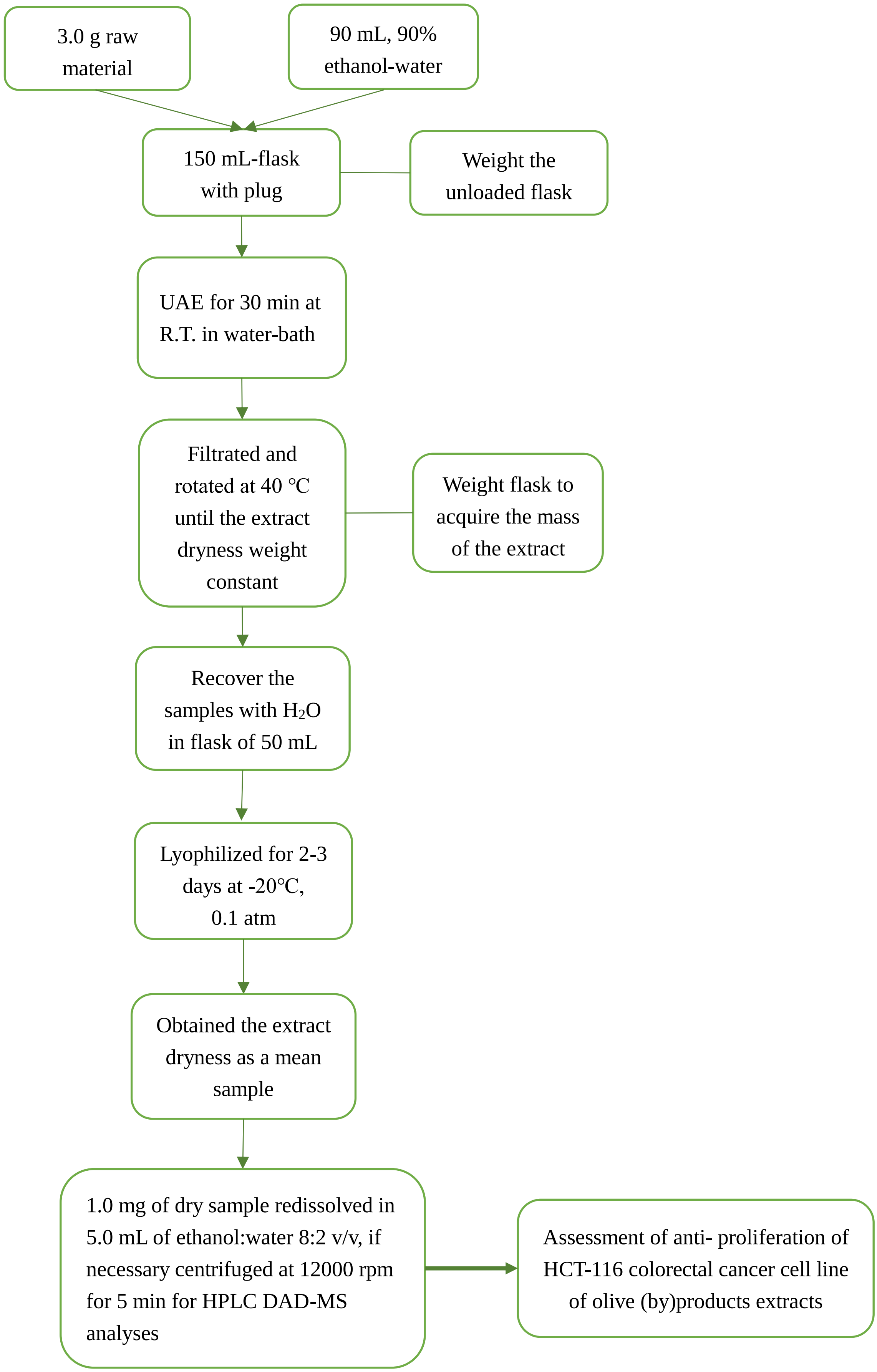

Figure S2. HPLC profile of triterpenoids for mixture standards: A, maslinic acid; B, oleanolic acid; C, ursolic acid; D, erythrodiol

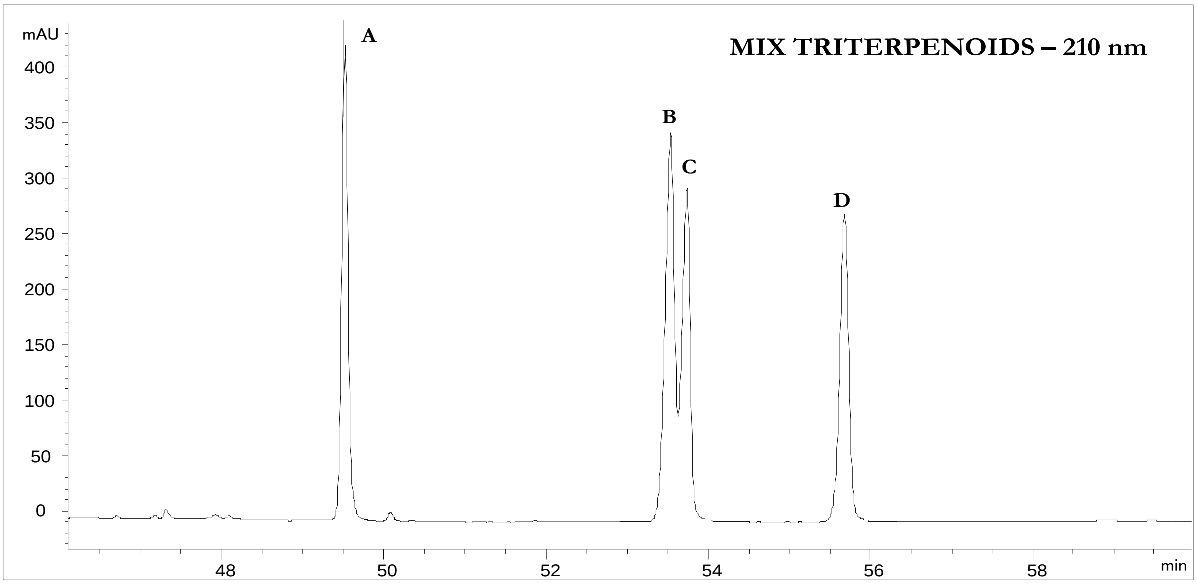

Supplement: Supplementary file 1 [file foods-10-02823-s001.zip › foods-1429655-supplementary.pdf]
